# Task Shifting the Management of Non-Communicable Diseases to Nurses in Kibera, Kenya: *Does It Work*?

**DOI:** 10.1371/journal.pone.0145634

**Published:** 2016-01-26

**Authors:** David Some, Jeffrey K. Edwards, Tony Reid, Rafael Van den Bergh, Rose J. Kosgei, Ewan Wilkinson, Bienvenu Baruani, Walter Kizito, Kelly Khabala, Safieh Shah, Joseph Kibachio, Phylles Musembi

**Affiliations:** 1 Médecins Sans Frontières, Nairobi, Kenya; 2 Médecins Sans Frontières, Operational Research Unit, Brussels, Belgium; 3 College of Health Sciences, Department of Obstetrics and Gynaecology, University of Nairobi, Kenya; 4 Chester University, Chester, United Kingdom; 5 Ministry of Health, Non Communicable Diseases Control Unit, Nairobi, Kenya; 6 Ministry of Health, Sub County Medical Officer of Health, Nairobi, Kenya; London School of Hygiene and Tropical Medicine, UNITED KINGDOM

## Abstract

**Background:**

In sub-Saharan Africa there is an increasing need to leverage available health care workers to provide care for non-communicable diseases (NCDs). This study was conducted to evaluate adherence to Médecins Sans Frontières clinical protocols when the care of five stable NCDs (hypertension, diabetes mellitus type 2, epilepsy, asthma, and sickle cell) was shifted from clinical officers to nurses.

**Methods:**

Descriptive, retrospective review of routinely collected clinic data from two integrated primary health care facilities within an urban informal settlement, Kibera, Nairobi, Kenya (May to August 2014).

**Results:**

There were 3,554 consultations (2025 patients); 733 (21%) were by nurses out of which 725 met the inclusion criteria among 616 patients. Hypertension (64%, 397/616) was the most frequent NCD followed by asthma (17%, 106/616) and diabetes mellitus (15%, 95/616). Adherence to screening questions ranged from 65% to 86%, with an average of 69%. Weight and blood pressure measurements were completed in 89% and 96% of those required. Laboratory results were reviewed in 91% of indicated visits. Laboratory testing per NCD protocols was higher in those with hypertension (88%) than diabetes mellitus (67%) upon review. Only 17 (2%) consultations were referred back to clinical officers.

**Conclusion:**

Nurses are able to adhere to protocols for managing stable NCD patients based on clear and standardized protocols and guidelines, thus paving the way towards task shifting of NCD care to nurses to help relieve the significant healthcare gap in developing countries.

## Introduction

Sub-Saharan Africa (SSA) is experiencing a demographic and epidemiological transition characterized by a rapidly growing urban population. This population is afflicted by both acute infections and chronic diseases, though management of acute infections still remains the main focus of most health systems in SSA. This is despite a progressive rise in non-communicable diseases (NCDs). The burden of NCDs such as hypertension (HT) and diabetes mellitus (DM) is overwhelming the existing health systems, particularly within informal settings. For instance, the prevalence of DM in Kibera, the largest informal settlement in Kenya, is 5.3%, which is higher than the national average of 4.2%[1] NCDs are now responsible for over 50% of all hospital admissions and deaths in Kenya, with projections to exceed communicable diseases as the leading cause of mortality by 2025.[2] The Kenyan populace is largely unscreened for NCDs; hence the burden is probably even higher.[3]

In Kenya, most NCDs are managed in tertiary health facilities, placing a workload strain on their staff. To overcome this challenge and improve access, Médecins Sans Frontières (MSF) and the Ministry of Health (MoH) introduced a model of managing NCDs in a primary health care (PHC) setting, by task shifting the care of stable NCD patients in the Kibera clinics to nurses. Task shifting is defined as the transfer of a task normally performed by a more highly trained health care worker (HCW) to another with a different, usually lower level of education and training.[4,5] This strategy has been recommended by the World Health Organization (WHO) for low and middle income countries, due to the low HCW to patient ratio.[4,5]

There is evidence that demonstrates the positive impact of task shifting of HIV care in these settings.[6,7,8] However, there is limited evidence for NCDs. One study from rural South Africa documented task shifting for HT, DM and asthma to nurses but with significant physician support. This study showed good outcomes, although with a small sample size (n = 224).[9] Another study from rural South Africa, described task shifting of DM only, and also had a small sample (n = 220).[10] In a third study from Cameroon, only HT was task shifted to nurses.[11] To the best of our knowledge, there are no studies from Kenya or SSA on task shifting five stable NCDs (and HIV), to nurses, or from an informal settlement context. In addition, none of the studies analyzed the ability of nurses to follow structured diagnosis and management protocols, which would otherwise be provided by clinical officers.

The aim of the study was to evaluate adherence to MSF clinical decision support protocols when the care of five NCDs (HT, DM, asthma, epilepsy and sickle cell disease (SCD)) was shifted from clinical officers to nurses in two integrated primary health care facilities in Kibera, Kenya from May to August 2014.

## Method

### Design

This was a retrospective review of routinely collected clinic data.

### Setting

Kenya is an east African country with a population of over 40 million.[12] It is experiencing an increasing burden of NCDs, including within informal settlements, putting a strain on the health care work force.[13] Kibera is an urban, informal settlement in the capital, Nairobi, with a population of about 240,000 people.[14] Kibera is characterized by poverty, and a lack of potable water, housing and access to health care, resulting in a large disease burden and poor health outcomes.[15] MSF, in collaboration with the MoH, offers free comprehensive PHC service, including NCD management.

### MSF Kibera NCD Programme

The MSF Kibera NCD programme started in 2009 in two PHC facilities, in response to an increasing number of NCD cases; there is an active cohort of 2,200 NCD and 5,500 HIV patients, leading to approximately 8,000 consultations per month. In these MSF facilities, integrated care includes acute and chronic services for HIV, TB, maternal-child health, NCDs, mental health, and sexual-gender based violence, all provided by the same clinical staff, who routinely rotate positions.

The healthcare team consists of a supervising physician, clinical officers, nurses, counselors, social workers, health promoters and laboratory staff, as described previously.[16] All routine NCD labs are run at the larger PHC facility. All services, including medication, are provided free of charge. Referrals are made to either Mbagathi District Hospital or Kenyatta National Hospital.

**Fig 1:**
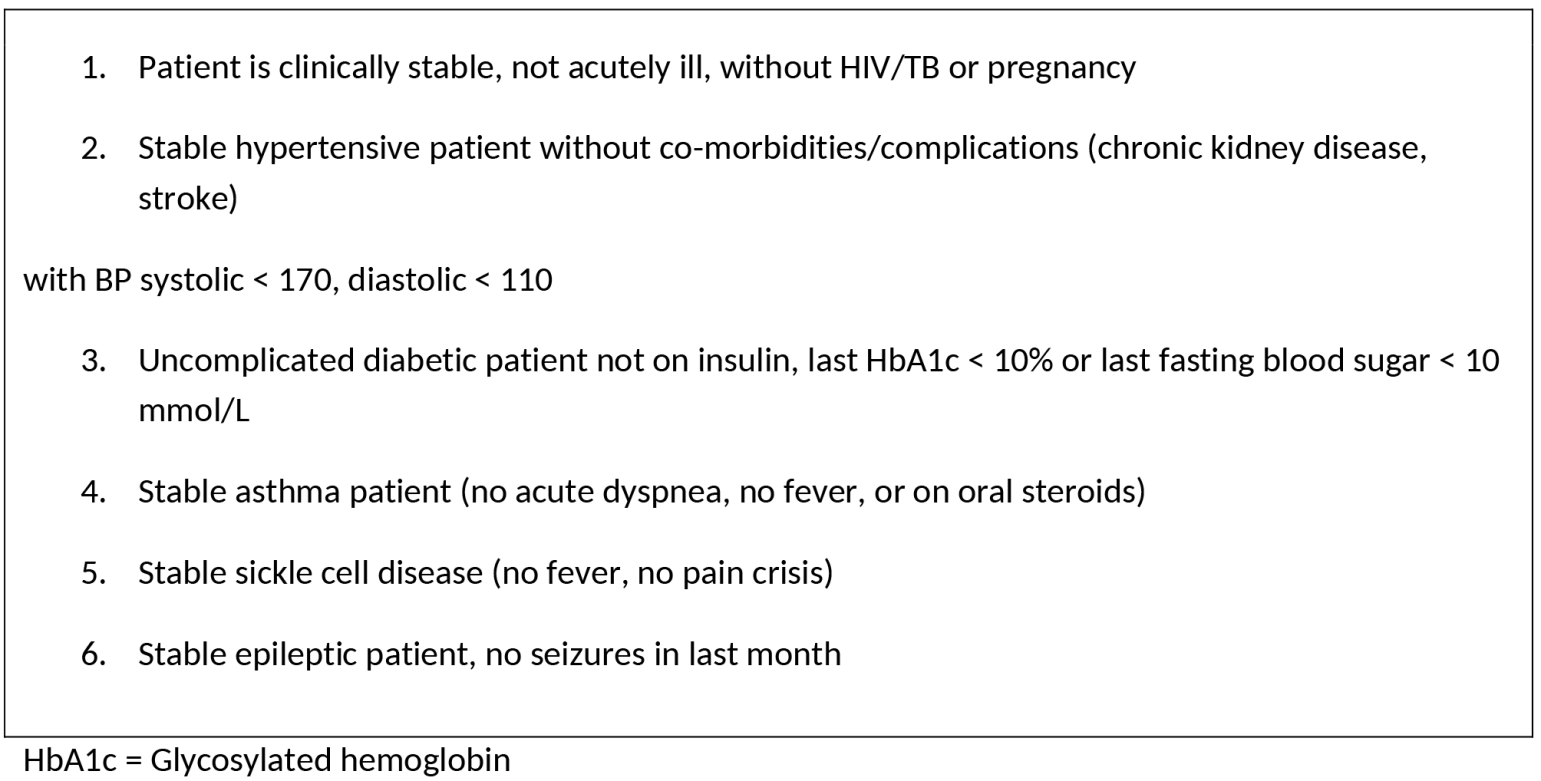
NCD patient inclusion criteria for nurse task shifting within primary care clinics in Kibera, Nairobi, Kenya (May-August 2014).

The volume of care translates into 45 to 50 consultations per clinical officer per day. Until early 2014, clinical officers managed all NCD cases. This resulted in an overwhelming NCD patient volume load. To overcome this challenge, management of some stable NCD cases was shifted to three nurses from March, 2014. The patient inclusion criteria for nurse task shifting are in Fig 1. The same nurses were already task shifting for stable patients with HIV, TB and general outpatient consultations.

Five conditions were task shifted to nurses: HT, DM, epilepsy, SCD and asthma. Within the NCD programme, the diagnostic criteria for HT were: two or more high BP measurements (>140/90) recorded during two or more clinic visits and for DM, fasting plasma glucose ≥7.0 mmol/l (126 mg/dl). A positive sickle cell test confirmed SCD. For asthma, the criteria included shortness of breath, presence of an identified asthma trigger and reversal of symptoms with an inhaled beta-agonist medication. Epilepsy was diagnosed through detailed medical history with confirmed witness account of repeated seizures (at least two, greater than 24 hours apart) that were not fever related.

Routine follow up laboratory testing for hypertension included creatinine clearance and fasting total cholesterol yearly, while diabetes also included HbA1c every 6 months. During clinical visits nurses were required to document whether they reviewed previous labs and ordered required laboratory tests per NCD protocol based upon date of last test.

All nurses selected for the NCD program were four-year academically trained in Kenya before joining MSF. Individually they had at least five years clinical nursing experience with MSF and were allowed to prescribe medications under Kenya regulations. Each nurse underwent standardized training on the five NCDs for one week with didactics and clinical case scenarios. In addition, they were trained using structured clinical decision support protocols, provided by the NCD supervising physician and included follow up field mentorship.

The NCD clinical decision support protocols and training were aligned with available MSF, Kenyan MoH and international guidelines as of 2013, for each of the five NCD diagnoses. The nurses worked within the PHC setting with continuous on-site clinical officer supervision and had NCD protocols available in both paper-bound form and electronically. The NCD trained nurses had no additional salary increase or incentives compared with other MSF/MOH nurses. There was no turnover of these nurses during the study period.

### Study Population

The study included all NCD patients (adults and children), who had consultations performed by the three trained nurses in the Kibera project between May and August 2014 based on the criteria defined for task shifting. Patients and consultations where a diagnosis was not recorded were excluded from the analysis.

### Data Collection, Management and Analysis

Data were collected to determine the number and proportion of NCD consultations where patients were screened for medication adherence, side effects and complications; blood tests requested and medications prescribed as per protocol; and referred back to clinical officers as needed. NCD consultations by nurse task shifters were recorded in a standardized paper format (available online). Clinical and laboratory data were retrieved from patient files using a structured data capture form, double entered into an Epi Data software and de-identified for confidentiality (version 3.1, EpiData Association, Odense, Denmark). All consultations done by the three nurses were compared against the variables in the protocols. Descriptive and summary statistics were used; analysis was done using Epi Info 7 Analysis software package (Centers for Disease Control, Atlanta, GA, USA).a Statistical significance was evaluated using chi-square and Fisher exact testing and utilized a two tailed p-value < 0.05.

### Ethics

For this study, routinely collected MSF data was retrospectively reviewed and de-identified. Hence, informed consent from patients or their families was not obtained. Ethics approval was obtained from the Kenya Medical Research Institute (Nairobi, Kenya) and the study met the Médecins Sans Frontières Ethics Review Board (Geneva, Switzerland) criteria for studies of routinely collected data and was also approved by the Ethics Advisory Group of the International Union Against Tuberculosis and Lung Disease (Paris, France).

## Results

During the study period, 3554 NCD consultations were conducted among 616 patients with 733 (21%) cumulative consultations by the task shifting nurses. Eight patients (1%) were excluded for lack of a recorded diagnosis, resulting in 725 consultations being analysed. Of these patients, 99% met the nurse task shifting inclusion criteria, 1% (13) cases were seen that were co-morbid HIV and NCD. Baseline characteristics are described in Table 1. The majority (72%, 445/616) of patients were female, (p = 0.05). Hypertension (64%, 397/616) was the most frequent NCD followed by asthma (17%, 106/616) and DM (15%, 95/616). There were 133/616 (22%) of patients with multiple co-morbid NCDs. Patients with HT and DM were older compared to those with asthma, epilepsy and SCD. Initial diagnosis and enrolment to NCD care (HT, DM or asthma) for 12 patients was completed by the nurses during routine outpatient visits.

**Table 1:** Socio-demographic and clinical characteristics of individual non-communicable disease patients at first nurse clinician consultation in two integrated primary health care clinics in Kibera, Kenya (May-August 2014).

Variables		Hypertension N (%)	Diabetes N (%)	Asthma N (%)	Sickle Cell N (%)	Epilepsy N (%)	Total N (%)
Primary NCD^1^ (%)		397 (64)	95 (15)	106 (17)	7 (1)	11 (2)	616 (100)
Comorbidity with other NCD		66 (11)	52 (8)	15 (2)	0	0	133 (22)
Sex	Male	94 (24)	25 (26)	31 (29)	3 (43)	6 (55)	159 (26)
	Female	298 (75)	67 (71)	73 (69)	3 (43)	4 (36)	445(72)
	Not recorded	5 (1)	3 (3)	2 (2)	1 (14)	1 (9)	12(2)
Median age, years (IQR)		50 (42–58)	51 (44–58)	38 (28–46)	11 (3–16)	15 (14–23)	
Median time in cohort, years (IQR)		3 (2–4)	3 (2–4)	2 (1–3)	2 (2–3)	2 (2–5)	
Initial enrollment in NCD cohort by nurse clinician		5 (1)	1 (1)	6 (5)	0	0	12 (2)
Median body mass index (IQR)		26 (23–30)	27 (23–30)	23 (20–25)	n/a	23 (19–30)	
Median systolic blood pressure (IQR)		135 (124–146)	130 (119–145)	118 (108–130)	n/a	115 (109–121)	
Median diastolic blood pressure (IQR)		82 (76–89)	81 (72–90)	75 (69–83)	n/a	72 (68–84)	
Median HbA1c (IQR)^2^		n/a	7.0 (6–8)	n/a	n/a	n/a	
Median total cholesterol (IQR)		4.9 (4–5)	4.9 (4–7)	n/a	n/a	n/a	

^1^Includes more than one NCD, see comorbidity row.

^2^Percent glycosylated haemoglobin.

IQR = interquartile, n/a = not applicable.

Weight measurements were completed in 89% of all NCD patients. For those with HT or DM, blood pressure readings were recorded in 96% of patients. Laboratory results were reviewed in 91% of HT and DM patients at the time of visit. Adherence to asking the three required screening questions (medication adherence, side effects and complications) varied from 65% for DM to 86% for SCD, with an average of 69%, but was not statistically significant between the groups. Appropriate laboratory testing was more consistent in those with HT than DM averaging 88% versus 67%, although there were only nine consultations for DM and differences were not statistically significant. Only 57% of patients with DM had an HbA1c ordered when indicated, but this result was affected by lack of reagents during part of the study period when fasting serum glucose was ordered instead.

Seventeen (2%) consultations were referred back to clinical officers. The reasons for referral were uncontrolled HT (3), uncontrolled DM (7), medication side effects (4), and NCD-associated complications (3). Adherences to the clinical protocols are described in Table 2. There were no reported mortalities among NCD patients seen by the task-shifting nurses during the study period.

**Table 2:** Adherence to MSF protocols among non-communicable disease consultations by nurse clinicians in two integrated primary health care clinics in Kibera, Kenya (May-August 2014).

Variables	HTN^1^ N (%)	Diabetes N (%)	Asthma N (%)	Sickle Cell N (%)	Epilepsy N (%)	Total N (%)
Total consultations	471 (65)	106 (15)	130 (18)	7 (1)	11 (1)	725 (100)
Patients asked about adherence, side effects, and complications	332 (70)	72 (68)	84 (65)	6 (86)	9 (82)	503 (69)
Weight checked	428 (91)	93 (89)	111 (86)	5 (71)	6 (55)	643 (89)
Blood pressure checked	458 (98)	98 (93)	n/a	n/a	n/a	556/577 (96)
Lab results reviewed	431 (92)	91 (86)	n/a	n/a	n/a	522/577 (91)
Creatinine ordered as per protocol	59/66 (89)	6/9 (67)	n/a	n/a	n/a	65/75 (86)
Cholesterol ordered as per protocol	58/66 (88)	6/9 (67)	n/a	n/a	n/a	64/75 (85)
HbA1c ordered as per protocol^2^	n/a	20/35 (57)	n/a	n/a	n/a	20/35 (57)
Medication altered at consultation:						
Medications added	9 (2)	7 (7)	0	0	0	16 (2)
Medication stopped	4 (1)	0	0	0	0	4 (1)
Referral back to clinical officer	10/471 (2)	7/106 (7)	0/130 (0)	0/7 (0)	0/11 (0)	17/725 (2)

^1^Hypertension.

^2^Glycosylated hemoglobin (HbA1c) test was only ordered when the reagent was available.

n/a = not applicable.

## Discussion

This is the first documented study in the sub-Saharan region, that describes and measures the process of task shifting five NCDs to nurses who also manage routine primary and HIV care, within an informal urban settlement. Our study shows an adherence to protocols and process indicators by nurses of 69% for routine screening questions and 81% for routine laboratory monitoring. Overall compliance with necessary weight measurements, blood pressure monitoring and laboratory review was greater than 90%. Lastly, there were very few referrals back to the clinical officers.

This study is unique in that it focuses on measuring the process of providing quality healthcare by task shifting nurses, rather than outcomes. Measuring the process of delivering care is frequently a more sensitive measurement of quality than outcomes because of the inherent variability in patient complexity can lead to confounding.[17] The measurement of outcomes would be best completed by having a randomized comparison group seen by the clinical officers. However, in our extremely resource-constrained context, the primary goal was off-loading more routine clinical visits from over-burdened clinical officers and improving care access, as had already been successfully done for HIV/TB patients within our program. This would also likely significantly improve patient flow and reduce patient waiting time, which had been previously estimated at 4–6 hours per patient (internal MSF review).

For those living within the highly impoverished community of an informal settlement, waiting extended periods of time for routine follow-up care of HIV and/or chronic diseases can translate into lost wages or potential employment opportunities. Given this context and extended previous experience with nurse task shifting for HIV/TB, it did not appear ethical to utilize a randomized control group design to obtain outcome measurements.

This study is also unique from another perspective in that it leverages an already existing HIV clinical platform and adds the care of NCDs on top, without the need for additional human resources. The same nurses (and clinical officers) providing care for HIV patients were trained to provide NCD care. In addition, the only further laboratory tests that were added to our routine HIV panel were glucose and HbA1c. Others have called for this approach, particularly for those HIV programs that are supported by the PEPFAR program.[18] It does not appear to be a significant leap from providing HIV care to NCD care, using the lessons learned and similar methodology.

The results from this study indicate that task shifting multiple NCDs to trained and supervised nurses using a structured clinical decision support protocol is achievable. It also implies that nurses are able to follow the process of clinical protocols for multiple different non-communicable diseases, sometimes comorbidly, despite their complexity. Although these results are an early assessment of the programme, they suggest that the training on the use of the protocols was adequate and easy to follow. They also indicate that it is safe to continue the task shifting programme.

There are no other studies directly comparable to ours. Most studies on this topic, task shifted only one NCD, unlike the five in Kibera.[19] Task shifting, as done in HIV care where it has been shown to be feasible, is the closest in terms of complexity.[19] There are multiple studies that demonstrate that nurses follow protocols appropriately and sometimes superiorly to physicians, but none that compare nurses directly to clinical officers.[6,20,21,22] It is likely that the Kibera nurses would perform comparably to clinical officers with similar patients if a randomized control group had been implemented.

Strengths of the study are the large sample size, a stable cohort of patients that met the study’s eligibility criteria, and rigorous data collection. It was conducted in a routine clinical setting, which suggests that the results reflect reality in that context. Some limitations are recognized. We were unable to directly compare the nurses’ adherence to protocols to that of clinical officers or clinical outcomes since they each managed patient cohorts with different degrees of complexity. In addition, it is possible that the nurses could have falsely reported completing some of the required tasks, and lack of referral back to a clinical officer does not exclude overlooked clinical indications that may have required referral. Other limitations were the inability to assess long-term patient outcomes and the short time period since programme implementation.

The results from this study are encouraging and the MSF/MoH clinic plans to expand the number of nurses trained for this purpose. This innovative model may be taken up by other primary health care facilities that are faced with tackling the increasing burden of NCDs.

## Conclusion

This study has demonstrated that nurses working within a resource-constrained, primary care and HIV setting, can successfully follow protocols managing stable patients with multiple NCDs. It is an early key finding considering the shortage of specialized clinicians and a rising burden of NCDs. This approach could be applied in other similar HIV-based programs to extend access to areas with increasing need of NCD care and limited resources. Further study needs to be completed to confirm adequate long-term outcomes are achievable and sustainable.

## Supporting Information

S1 Fig NTS dataset PLOS: Nurse Task shifting data set that was used for the analysis and findings of the study. (XLSX)

S2 Fig NTS Data Collection Tool: a standardized paper format that was used for data collection by the task shifting nurses. (PDF)
