# Supplementary material for: Task Shifting the Management of Non-Communicable Diseases to Nurses in Kibera, Kenya: Does It Work?
Source: PLoS One. 2016 Jan 26;11(1):e0145634. doi: 10.1371/journal.pone.0145634 (PMC4727908; doi:10.1371/journal.pone.0145634)
Supplement: S2 Fig — (PDF) [file pone.0145634.s002.pdf]

Nurse Task Shifting Data Collection Tool

Clinic: Kibera South ☐ Silanga ☐

Nurse's name: ______________________________

Date of Visit: _____ / _____ / _____
dd / mm / yy

PATIENT DETAILS

NCD Number: ____________________________ New Visit ☐ Follow Up Visit ☐

Sex: Male ☐ Female ☐ Patient Age :___________

MEDICAL HISTORY

HTN ☐ DM ☐ Asthma ☐ Epilepsy ☐ Sickle Cell ☐ HIV comorbid: Yes ☐ No ☐

CURRENT MEDICAL STATUS

Triaged with any of following?: SOB: ☐ Fever > 38.0: ☐ Sickle Cell Crisis: ☐ Seizure in the last month: ☐

Any complications reported?: Yes ☐ No ☐ Patient adherent to meds?: Yes ☐ No ☐ Any side effects reported?: Yes ☐ No ☐

Patient Wt:_______ Ht:________ SBP:_____ DBP:_____

Date of last labs:______________________

Results: HbA1c results:________ Creatinine:________ CrCl:________ Total Cholesterol:________ Fasting glucose:________

LAB AND MEDICATION PROTOCOL

Creatinine ordered: ☐ Cholesterol ordered: ☐ HbA1c ordered: ☐ UA ordered: ☐ Fasting glucose ordered: ☐

New NCD patient started on medication?: ☐ New drug added during follow up to improve control?: ☐

Drug stopped secondary side effects? : ☐

CLINICAL OFFICER CONSULTATIONS

Patient referred back to clincal officer for consultation? ☐

Reasons for referral: Uncontrolled: ☐ Medication side effects: ☐ Medical complication: ☐ New Cormorbidity ☐

Other reason:____

MEDICATION ADHERENCE CLUBS

Meets Medication Adherence Club criteria? Yes ☐ No ☐

Patient agrees to MAC referral? Yes ☐ No ☐

Patient delcines MAC referral: Because of time of day: ☐ Because of day of week: ☐ Because prefers individual appt: ☐

MAC referral criteria: 1. age ≥ 25 years old 2. > 6 months on medication 3. BP < 150/100 4. HbA1c < 8.0% (if diabetic)
